# Metabolite and gut microbiota co-biomarkers in Danggui Shaoyao San: insights into a shared therapeutic approach

**DOI:** 10.3389/fphar.2025.1698734

**Published:** 2026-01-12

**Authors:** Xin Fu, Dinghan Peng, Yang Yu, Mingguo Cao, Xin Zheng, Songquan Wu

**Affiliations:** 1 School of Medicine, Lishui University, Lishui, Zhejiang, China; 2 Affiliated Hospital of Integrated Traditional Chinese and Western Meicine, Nanjing University of Chinese Medicine, Nanjing, Jiangsu, China

**Keywords:** Alzheimer disease (AD), bacteroidetes, Danggui Shaoyao San (DSS), phenylalanine, polycystic ovary syndrome (PCOS), tryptophan, type 2 diabetes mellitus(T2DM)

## Abstract

Danggui Shaoyao San (DSS), a classical multi-herbal formulation of traditional Chinese medicine, demonstrates therapeutic potential for Type 2 Diabetes Mellitus (T2DM), Alzheimer’s Disease (AD), and Polycystic Ovary Syndrome (PCOS). This review proposes a unified mechanism whereby DSS exerts its effects by modulating a network of shared pathological biomarkers across these disorders. We identify tryptophan (Trp) and phenylalanine (Phe) as host-derived metabolic biomarkers in plasma, and the gut bacterial phylum Bacteroidetes as a shared gut microbial biomarker. The therapeutic actions of DSS are mediated by its botanical constituents (e.g., ligustilides, paeoniflorin, ferulic acid), which help correct the dysregulated Trp-kynurenine and Phe metabolic pathways, while simultaneously enriching Bacteroidetes to alleviate gut dysbiosis and rebalance the gut-brain axis. This coordinated regulation of shared host metabolic and gut microbial biomarkers provides a scientific rationale for applying DSS as a multi-targeted agent, illustrating a molecular basis for a shared therapeutic approach.

## 1 Introduction

Type 2 Diabetes Mellitus (T2DM), characterized by insulin resistance, is increasingly linked to the pathophysiology of both Alzheimer’s Disease (AD) and Polycystic Ovary Syndrome (PCOS) through shared mechanisms involving insulin signaling dysregulation (Society, 2021; Xurui et al., 2024; Wu et al., 2025). This convergence of metabolic dysfunction suggests a potential rationale for exploring shared therapeutic strategies.

Danggui Shaoyao San (DSS) was first recorded in the classic text Jin Gui Yao Lue and was originally indicated for “various abdominal pains in women.” In traditional Chinese medicine (TCM) theory, its therapeutic basis extends to liver and kidney disorders through the principle of harmonizing the Liver and Spleen and nourishing Blood to emolliate the Liver. This foundational theory posits that DSS addresses underlying patterns of blood deficiency and stagnation, which are conceptually linked to modern manifestations of metabolic dysregulation and neuroendocrine imbalance. In the development of modern medicine, its clinical applications have gradually expanded to include diseases such as liver and kidney disorders like liver cirrhosis, neurological conditions like Alzheimer’s Disease (AD), and skin diseases like melasma (Author Anonymous, 2024). The formulation is composed of six botanical drugs.:*Angelica sinensis* (Oliv.) Diels (Family: Apiaceae; Drug: Angelicae Sinensis Radix),* Paeonia lactiflora* Pall. (Family: Paeoniaceae; Drug: Paeoniae Radix Alba),* Ligusticum sinense ‘Chuanxiong’* (Family: Apiaceae; Drug: Chuanxiong Rhizoma),* Atractylodes macrocephala* Koidz. (Family: Asteraceae; Drug: Atractylodis Macrocephalae Rhizoma),* Alisma plantago-aquatica subsp. orientale* (Sam.) Sam. (Family: Alismataceae; Drug: Alismatis Rhizoma),* Poria cocos* (Schwein.) F.A.Wolf (Family: Polyporaceae; Drug: Poria)in a ratio of 3: 10: 4: 5: 4: 3. The main producing areas of *A. sinensis* (Danggui) are Gansu Province and Yunnan Province in China (REN and DENG, 2023). *P. lactiflora* (Shaoyao) is primarily from Bozhou in Anhui, Zhongjiang in Sichuan, Pan’an in Zhejiang, and Heze in Shandong (Xia and o, 2023). *Poria cocos* (Fuling) is mainly distributed in Luotian in Hubei, Yuexi in Anhui, and Jinggu in Yunnan (Yunlu, 2024). *Alisma plantago-aquatica* (Zexie) is found in Jiangxi, Sichuan, and other regions in China (Author Anonymous, 2022). *Atractylodes macrocephala* (Baizhu) is mostly cultivated in Pan’an in Zhejiang and Bozhou in Anhui (LIU Ya-dong et al., 2024). *Ligusticum chuanxiong* (Chuanxiong) is distributed in Sichuan, Yunnan, and Guizhou in China (Wu et al., 2025). Their botanical characteristics are shown in Supplementary Table S1. It is worth noting that *P. cocos*, as a fungus, does not belong to the category of plants.

Modern pharmacological studies have provided a mechanistic basis for expanding the clinical use of DSS in diabetic complications. Integrated UHPLC-Q Exactive-Orbitrap-MS and network pharmacology analyses of DSS drug-containing serum identified 39 bioavailable metabolites. Notably, the ligustilides common to *A. sinensis* and *L. chuanxiong:*senkyunolide A and Z-ligustilide, have been shown to activate the AMPK/PINK1 pathway while suppressing the TLR4–NF-κB cascade, thereby markedly reducing high-glucose-induced oxidative stress (ROS) and pro-inflammatory cytokines (IL-6, TNF-α) (Huang et al., 2025). The *P. lactiflora*-specific monoterpene glycoside paeoniflorin improves mitochondrial membrane potential and inhibits caspase-3/9 activity via the adenosine A1 receptor–CREB axis, exerting anti-apoptotic and axon-regenerative effects in neurons injured by 27-hydroxycholesterol or amyloid-β (Hong et al., 2022). Furthermore, ferulic acid, primarily from *L. chuanxiong* (with contributions from *A. macrocephala* and *Alisma orientale*), attenuates mesangial hypertrophy and basement-membrane thickening by inhibiting the TGF-β/Smad and p38 MAPK signalling pathways (Wang et al., 2025). While these findings confirm the broad anti-inflammatory and anti-oxidative activities of DSS and its constituents, their specific protective effects and mechanisms against diabetic neuropathy and nephropathy constitute a critical area for future investigation to fully elucidate DSS’s therapeutic potential.

This pharmacological profile is supported by clinical evidence. In T2DM, clinical studies have demonstrated that DSS not only improves glycemic control but also alleviates specific complications, including diabetic neuropathy and nephropathy (Xiao-bin, 2019; Material et al., 2022). Beyond metabolic disorders, a systematic review and meta-analysis involving over 500 participants confirmed its significant benefits in improving cognitive function in patients with AD (Haifang, 2023; Kim and Cho, 2020). Furthermore, in the management of PCOS: a condition intrinsically linked to insulin resistance,DSS intervention has been shown to ameliorate hyperandrogenism, promote ovarian function, and restore menstrual cyclicity (Zhang and shelley, 2023; Yang et al., 2020). Collectively, this convergent clinical evidence from three distinct disease domains underscores the potential of DSS as a “shared therapeutic” agent targeting the common node of insulin resistance and its systemic manifestations.

Chromatographic fingerprinting has verified the chemical consistency of DSS preparations, establishing a reliable basis for its pharmacological study (Liu et al., 2020). Metabolomic analyses further reveal that DSS alleviates conditions like dysmenorrhea by modulating key metabolites, including tryptophan and phenylalanine (Bauermeister et al., 2022; Li N. et al., 2022). Complementing this, gut microbiota profiling shows that DSS intervention consistently enriches Bacteroidetes and improves the Bacteroidetes/Firmicutes ratio, suggesting a microbial mechanism underlying its efficacy in memory-impairment and metabolic models (Liu P. et al., 2022; Wang et al., 2024). Collectively, these findings support the hypothesis that DSS may mitigate AD, T2DM, and PCOS through coordinated regulation of tryptophan and phenylalanine pathways and restoration of Bacteroidetes abundance.

Although TCM is characterized by multi-target and multi-component effects, its specific mechanisms often remain incompletely understood. To bridge this knowledge gap, this review aims to first identify common pathological targets (key biomarkers and gut microbiota) across AD, T2DM, and PCOS, and then critically evaluate the evidence for how DSS and its active constituent precisely interact with them.

## 2 Metabolite biomarkers in T2DM, AD, PCOS

**FIGURE 1:**
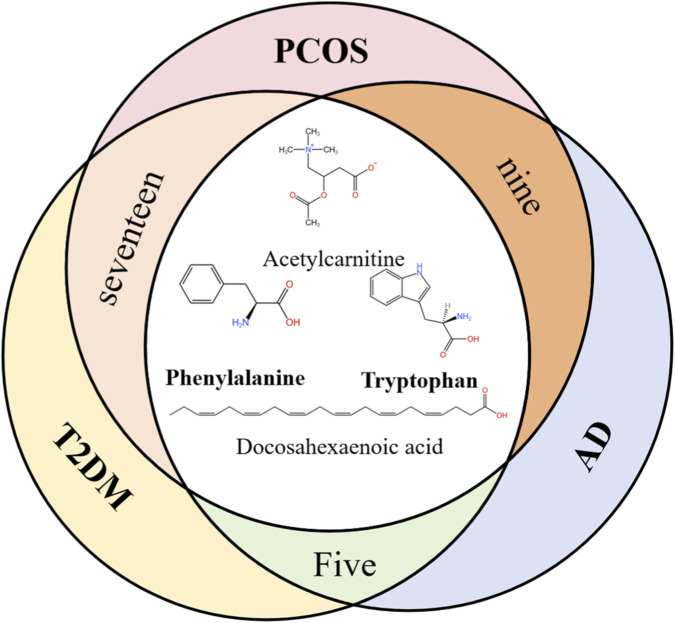
The metabolite co-biomarkers among the three comorbidities. (There are 17 common metabolites between PCOS and T2DM, 9 common metabolites between AD and PCOS, and 5 common metabolites between AD and T2DM).

Biological markers are diagnostic tools that reflect the pathological conditions of the human body and are measured from body fluids (Yule et al., 2024). In the blood, markers can be further divided into serum markers and plasma markers. After consulting a large number of documents from PubMed, we have collated the metabolite markers in three diseases: T2DM, AD, and PCOS into tables (seen Supplementary Tables S2–4). From this, we selected metabolites that are common to at least two of the three diseases or present in all three diseases, and compiled them into Table 1. Based on this table, we have drawn a Venn diagram. In the intersection of the Venn diagram, it is clearly visible that tryptophan and phenylalanine are the common biological markers for T2DM, AD, and PCOS (seen Figure 1).

**TABLE 1:** The metabolite co-biomarkers in T2DM, AD and PCOS.

Metabolite/Biomarker	Location	Change in T2DM	Change in AD	Change in PCOS	References
Propionylcarnitine (C3)	Plasma	↓	↓	​	Abu Bakar and Sarmidi (2017), Mapstone et al. (2014)
Arachidonic acid	Plasma	↑	↑	​	Abu Bakar and Sarmidi (2017), Tan et al. (2023)
Glucose	Plasma, Urine	↑	↑	​	Gogna et al. (2015), Yilmaz et al. (2020)
Lysophosphatidylcholine (LPC)	Plasma	↑	↓	​	Mapstone et al. (2014), Mastrangelo et al. (2016), Zhang et al. (2020a), He et al. (2021)
Phosphatidylcholine (PC)	Follicular fluid, Plasma	↓	↓	​	Mapstone et al. (2014), Zhang et al. (2020a), Zhu et al. (2011)
Glycine	Plasma	↓	​	↓	Merino et al. (2018), Okekunle et al. (2017), Ye et al. (2022)
Valine	Plasma, Serum	↑	​	↑	Abu Bakar and Sarmidi (2017), Gogna et al. (2015), Okekunle et al. (2017), Ramzan et al. (2022), Zhao et al. (2021)
Isoleucine	Plasma, Serum	↑	​	↑	Abu Bakar and Sarmidi (2017), Gogna et al. (2015), Okekunle et al. (2017), Ye et al. (2022), Ramzan et al. (2022)
Glutamic acid	Plasma, Serum	↑	​	↑	Okekunle et al. (2017), Ye et al. (2022), Zhao et al. (2021), Ding et al. (2022)
Alanine	Plasma, Serum	↑	​	↑	Abu Bakar and Sarmidi (2017), Okekunle et al. (2017), Ye et al. (2022)
Lysine	Plasma, Serum	↑	​	↑	Gogna et al. (2015), Okekunle et al. (2017), Ye et al. (2022)
Leucine	Plasma, Serum	↑	​	↑	Abu Bakar and Sarmidi (2017), Gogna et al. (2015), Okekunle et al. (2017), Ye et al. (2022), Ramzan et al. (2022)
Ornithine	Plasma, Serum	↑	​	↑	Okekunle et al. (2017), Zhao et al. (2021)
Histidine	Plasma, Serum	↑	​	↑	Gogna et al. (2015), Okekunle et al. (2017), Ye et al. (2022)
Glutamine	Plasma, Serum	↓	​	↓	Abu Bakar and Sarmidi (2017), Okekunle et al. (2017), Sun et al. (2012)
Asparagine	Plasma, Serum	↓	​	↑	Okekunle et al. (2017), Ye et al. (2022)
Palmitic acid	Follicular fluid, Plasma	↑	​	↑	Abu Bakar and Sarmidi (2017), Chen et al. (2016), Liu et al. (2022b)
Linoleic acid	Plasma, Serum	↑	​	↑	Abu Bakar and Sarmidi (2017), Dong et al. (2015)
Pyruvate	Follicular fluid, Plasma	↑	​	↑	Abu Bakar and Sarmidi (2017), Liu et al. (2022b)
Lactate	Plasma	↑	​	↑	Gogna et al. (2015), Sun et al. (2012)
Choline	Plasma	↑	​	↓	Gogna et al. (2015), Sun et al. (2012)
Threonine	Plasma	↑	​	↑	Gogna et al. (2015), Ye et al. (2022)
Ceramides (Cers)	Plasma, Serum	​	↑	↑	Zhang et al. (2020a), Sun et al. (2018), Li et al. (2019)
Triacylglycerols (TGs)	Follicular fluid, Plasma	​	↑	↑	Zhang et al. (2020a), Ban et al. (2021)
Free Fatty Acids	Follicular fluid, Plasma	​	↓	↑	Zhang et al. (2020a), Zhao et al. (2021), Sun et al. (2018), Zhao et al. (2014)
Taurocholic Acid (TCA)	Follicular Fluid, Brain	​	↓	↑	Pan et al. (2017), Koike et al. (2021), Yang et al. (2021a)
Lithocholic acid (LCA)	Follicular fluid, Plasma	​	↑	↓	Liu et al. (2022b), Koike et al. (2021)
Bile Acids	Follicular fluid, Plasma	​	↑	↑	Yang et al. (2021a), Shao et al. (2020)
Chenodeoxycholic Acid	Follicular fluid, Plasma	​	↑	↑	Yang et al. (2021a), Shao et al. (2020)
L-Carnitine	Follicular fluid, Plasma	​	↓	↑	He et al. (2021), Chen et al. (2020)
Glycerophosphocholine	Plasma, Serum	​	↑	↑	Ding et al. (2022), Sun et al. (2018)
Tryptophan	Plasma, Serum	↑	↑	↑	Mastrangelo et al. (2016), Okekunle et al. (2017), Zhao et al. (2021), Sun et al. (2018)
Phenylalanine	Plasma	↑	↑	↑	Abu Bakar and Sarmidi (2017), Tan et al. (2023), Gogna et al. (2015), Merino et al. (2018), Okekunle et al. (2017), Zhao et al. (2021)
Acetylcarnitine (C2)	Plasma, Serum	↑	↓	↓	Abu Bakar and Sarmidi (2017), He et al. (2021), Ding et al. (2022)
Docosahexaenoic acid (DHA)	Plasma, Serum	↓	↓	↓	Tan et al. (2023), Mastrangelo et al. (2016), Mousa et al. (2022)
Sphingomyelin	Plasma	↓	↑	↑	Abu Bakar and Sarmidi (2017), Zhang et al. (2020a), Sun et al. (2012), Sun et al. (2018)

Note on biomarker selection: The metabolites tryptophan and phenylalanine were prioritized as core co-plasma biomarkers for detailed discussion in this review. This selection was based on their established central role in the gut-brain axis and the availability of direct mechanistic evidence linking their metabolic pathways to the pharmacological actions of DSS. While other metabolites, such as docosahexaenoic acid (DHA), are altered in the comorbidities and have significant biological roles, their association with the specific gut microbiota-host co-metabolism and DSS-focused mechanisms examined herein is less directly characterized in the current literature.

### 2.1 Tryptophan as a co-biomarker in T2DM, AD and PCOS

Tryptophan is one of the essential amino acids necessary for protein synthesis, which metabolized mainly through kynurenine and serotonin pathways. In normal physiological conditions, more than 95% of tryptophan is metabolized by kynurenine pathway and the rest is converted to serotonin by intestinal chromaffin cells (Savitz, 2020). More and more studies reported that tryptophan metabolic dysfunction is associated with the occurrence and development of many diseases, such as T2DM, metabolic syndrome, inflammation, cancer, etc. (Benech et al., 2022; Priyadarshini et al., 2022).

#### 2.1.1 Tryptophan related to T2DM

Based on the tryptophan metabolic pathway, some studies have found that the ratio of plasma kynurenine to tryptophan is also directly correlated with insulin resistance (Oxenkrug et al., 2013) and T2DM (Rebnord et al., 2017), indicating that tryptophan metabolites may plays an important role in the pathogenesis of T2DM. Indolepropionic acid is also a deamination product of amino acid tryptophan (Menni et al., 2019), which may plays a protective role in T2DM by protecting β cell’s function (de Mello et al., 2017). Dietary restriction of tryptophan has been shown to modulate metabolic hormones in obese rats. A low-tryptophan diet reduced fasting circulating levels of glucose, insulin, C-peptide, and leptin, while concomitantly increasing levels of glucagon, pancreatic polypeptide, and glucagon-like peptide-1 (Zapata et al., 2018). This interplay between tryptophan intake and metabolic regulation is further supported by clinical observations. For instance, Roux-en-Y gastric bypass (RYGB) surgery reduces serum levels of tryptophan and its downstream metabolite kynurenine in patients with (T2DM), an effect potentially associated with improved glycemic control (HbA1c) and reduced body mass index (BMI). Importantly, obesity-driven chronic systemic inflammation is a key setting in which tryptophan-kynurenine (TRP-KYN) pathway metabolites are believed to play a mechanistic role, contributing to the development of obesity-related comorbidities including T2DM (Yeung et al., 2022). After clinical administration of metformin, insulin sensitivity increased along with downregulation of Kynurenine pathway (Muzik et al., 2017). These findings suggest that pancreatic 5-HT plays an important role in insulin secretion by acting on different 5-HT receptors expressed on different cell types. In the serotonin pathway of tryptophan, human beta cells produce and secrete serotonin in response to increased glucose concentration.5-HT decreased glucagon secretion and cAMP levels in adjacent α cells. This suggests that pharmacological activation of the 5-HT1F receptor by 5-HT reduces glucagon secretion, thereby exerting a hypoglycemic effect in diabetic mice (Almaça et al., 2016).

#### 2.1.2 Tryptophan related to AD

Impaired clearance of amyloid-beta (Aβ) peptide is a major pathophysiological factor in AD (Murphy and LeVine, 2010). In this context, endogenous tryptophan metabolites are increasingly recognized for their regulatory roles. For instance, 5-hydroxyindoleacetic acid (5-HIAA) and kynurenic acid (KYNA) can stimulate the activity/expression of neprilysin (NEP), an Aβ-degrading enzyme, thereby counteracting Aβ-induced neurotoxicity. Furthermore, various tryptophan metabolites modulate brain Aβ levels under both normal and pathological conditions by interacting with the aryl hydrocarbon receptor (AhR) and regulating downstream metalloproteinases (Maitre et al., 2020). It is noteworthy that alterations in tryptophan metabolism may be compartmentalized; LC-MS/MS quantification has revealed that changes in peripheral levels of serotonin (5-HT) and norepinephrine (NE) do not uniformly reflect those in the brain (Wang et al., 2019). Collectively, these mechanisms through which tryptophan metabolism influences Aβ dynamics contribute significantly to the pathogenesis and progression of AD.

#### 2.1.3 Tryptophan related to PCOS

Previous studies have demonstrated abnormalities in tryptophan catabolism in PCOS (Zhao et al., 2012; Refaey et al., 2017; Onesti et al., 2019; Min et al., 2020; Zangeneh et al., 2020). These findings suggest that the tryptophan-kynurenine pathway may be activated in PCOS pathogenesis. Specifically, the urinary kynurenine-to-tryptophan ratio showed a significant positive correlation with levels of key reproductive hormones:including luteinizing hormone (LH), the LH/follicle-stimulating hormone (FSH) ratio, anti-Müllerian hormone (AMH), and dehydroepiandrosterone sulfate (DHEAS), but not with FSH alone. Concurrently, metabolites such as 3-hydroxykynurenine (3-OH-KYN), quinolinic acid, the kynurenine-to-tryptophan ratio, and 3-hydroxyanthranilic acid (3-OH-AA) were positively correlated with measures of glucose metabolism and insulin resistance: fasting blood glucose (FBG), fasting serum insulin (FSIns), and the homeostatic model assessment of insulin resistance (HOMA-IR). These associations indicate a close link between tryptophan-kynurenine metabolism and both reproductive endocrine and metabolic dysregulation in PCOS (Yang et al., 2022; Han et al., 2025; Belenkaia et al., 2019).

As Kynurenine pathway is positively correlated with BMI, there is still an interaction between the pathophysiological mechanism of PCOS and abnormal tryptophan metabolism after excluding the influence of obesity. The levels of tryptophan, canurine and uric acid in all subjects were positively correlated with LH and AMH. Therefore, abnormal activation of the tryptophan-canurine pathway may affect neuroendocrine feedback in patients with PCOS, which may be a potential therapeutic target for PCOS. The study showed an increase in C-reactive protein (CRP), and an increase in IL-6 and TNF-α, in women with PCOS compared with the control group, and this increase was not associated with obesity (Rostamtabar et al., 2021). There is little information on the relationship between plasma CRP and tryptophan catabolism. To investigate this mechanism, a study employed an experimental endotoxemia model induced by intravenous lipopolysaccharide injection to examine the kynurenine pathway (KP). Enzyme activity analyses revealed that the activity of kynurenine 3-monooxygenase (KMO) and kynureninase (KYNU), along with kynurenine aminotransferase (KAT), increased within 3–6 h post-injection, leading to the depletion of tryptophan and kynurenine (Millischer et al., 2021). More relevant to the pathophysiology under discussion, the activity of indoleamine 2,3-dioxygenase 1/tryptophan 2,3-dioxygenase (IDO-1/TDO2) was upregulated at 24–48 h. This increase coincided with a peak in both the serum kynurenine-to-tryptophan (KYN/TRP) ratio and C-reactive protein (CRP) levels. Since IDO activity is known to be induced by low-grade inflammation and psychological stress, and given a recent finding of its negative correlation with hormone receptor activity (Onesti et al., 2019), we hypothesize that elevated pro-inflammatory cytokines, such as CRP, may drive the observed increase in IDO activity. Supporting this, plasma CRP levels strongly correlate with other key inflammatory markers like interleukin-6 (IL-6) and tumor necrosis factor-alpha (TNF-α), reliably reflecting systemic inflammatory activity (Felger et al., 2020).

### 2.2 Phenylalanine as a co-biomarker in T2DM, AD and PCOS

In recent years, phenylalanine metabolic dysregulation has become a hotspot in medical research. We searched many materials,and studies have revealed that abnormally elevated blood levels of phenylalanine are closely associated with several major diseases, including T2DM, AD, and PCOS (Zhou et al., 2022; Muri et al., 2024; Paczkowska et al., 2023). It is considered a potential “co-biomarker” that links metabolic, neuroendocrine, and reproductive health, providing important clues for understanding the common pathogenesis of these diseases and developing novel therapeutic strategies.

#### 2.2.1 Phenylalanine related to T2DM

Beyond branched-chain amino acids, the aromatic amino acid phenylalanine has emerged as a significant biomarker in the pathogenesis of T2DM. Epidemiological studies consistently identify elevated circulating phenylalanine as a robust predictor of future insulin resistance and T2DM, an association observed across diverse ethnicities and largely independent of obesity measures (Würtz et al., 2013; Tillin et al., 2015; Tai et al., 2010). Clinically, the close link between phenylalanine and diabetic metabolism is further evidenced by the finding that its plasma levels decrease in response to hypoglycemic agents like metformin (Preiss et al., 2016).

The pathophysiological role of phenylalanine extends beyond a mere biomarker. It actively contributes to insulin resistance through multiple, converging mechanisms. Experimental evidence indicates that phenylalanine and its metabolites can impair insulin signaling in skeletal muscle, potentially via pathways involving mTOR, JNK, and IRS1, leading to inhibited glucose transport/phosphorylation and reduced glycogen synthesis (Krebs et al., 2002; Dornhorst, 2001). Furthermore, phenylalanine-induced oxidative stress may inhibit phenylalanine hydroxylase activity, creating a feed-forward loop that elevates phenylalanine while reducing its conversion to tyrosine (Stephens et al., 2015). This metabolic dysregulation is compounded by hormonal crosstalk; for instance, hyperglucagonemia, common in T2DM,can shift phenylalanine metabolism toward oxidation, altering its bioavailability (Tessari et al., 1996).

This mechanistic understanding illuminates potential therapeutic strategies. Interventions that lower phenylalanine levels or modulate its metabolism show promise. For example, the gut-microbiota-modulating agent berberine alleviates diabetic symptoms partly by reducing aromatic amino acid metabolism (Yao et al., 2020). Interestingly, endogenous exercise-induced metabolites like N-lactoyl-phenylalanine (N-lac-phe) can deplete phenylalanine, offering a molecular explanation for physical activity’s protective effect against T2DM (Newgard et al., 2009; Robinson et al., 2014). Even pharmaceutical approaches utilize this pathway, as demonstrated by the phenylalanine-derived drug nateglinide, which enhances glucose-stimulated insulin secretion (Li VL. et al., 2022). Collectively, these insights position phenylalanine not only as a diagnostic marker but also as a node linking metabolic dysfunction, hormonal imbalance, and potential therapeutic intervention in T2DM.

#### 2.2.2 Phenylalanine related to AD

Emerging evidence positions phenylalanine at the intersection of metabolic dysregulation and protein aggregation in AD. Metabolomic analyses of *postmortem* human brain tissues reveal that phenylalanine levels are significantly upregulated in AD patients compared to controls, suggesting a systemic metabolic alteration associated with the disease (Liu et al., 2021). Beyond its role as a metabolite, phenylalanine residues within the amyloid-β (Aβ) peptide play a critical structural role in driving its pathogenic aggregation. Mechanistic studies demonstrate that the aromatic side chain of phenylalanine is crucial for Aβ self-assembly. For instance, substituting phenylalanine residues in Aβ with cyclohexylalanine (Cha)—a non-aromatic analog—effectively inhibits the formation of mature Aβ fibrils (Genji et al., 2017). Conversely, strategic mutation of Phe20 to Cha in Aβ-derived peptides can promote and stabilize the formation of neurotoxic oligomers, which are considered key pathogenic species in early AD (Haerianardakani et al., 2020). Collectively, these findings indicate a dual pathogenic link: aberrant elevation of brain phenylalanine may reflect or contribute to a metabolic environment conducive to AD pathogenesis, while the intrinsic properties of phenylalanine residues directly facilitate the aggregation of Aβ into toxic assemblies.

#### 2.2.3 Phenylalanine related to PCOS

Elevated circulating phenylalanine levels are not only a metabolic hallmark of PCOS but also implicated in its reproductive and neuroendocrine dysfunction. As a potential “warning sign” for compromised oocyte developmental competence (Huang et al., 2021), high phenylalanine may reflect a suboptimal follicular fluid environment, aligning with the established view that amino acid balance is crucial for blastocyst development (Gardner, 1998). More broadly, phenylalanine metabolism intersects with PCOS pathogenesis through its link to insulin resistance, a key driver of the syndrome. Intriguingly, phenylalanine is directly incorporated into arginine-phenylalanine-amide (RFamide)-related peptide-3 (RFRP3), a neuropeptide encoded by the Rfrp gene. RFRP3 acts in the mammalian hypothalamus to inhibit gonadotropin-releasing hormone (GnRH) secretion (Shaaban et al., 2018). Thus, perturbations in phenylalanine availability could theoretically influence the synthesis or function of RFRP3, potentially contributing to the dysregulated GnRH pulsatility and subsequent hormonal imbalances (e.g., elevated LH) characteristic of PCOS. This integrative perspective positions phenylalanine as a metabolic node connecting systemic insulin resistance, local ovarian follicle health, and central neuroendocrine regulation in PCOS.

## 3 Gut biomarkers in T2DM, AD and PCOS

The gut microbiota is a microbial community that colonizes the human body, comprising bacteria, fungi, archaea, viruses, and protozoa, and is regarded as an indispensable component of the human organism (Adak and Khan, 2019). Most studies suggest that, due to the sterile nature of the uterus, infants acquire gut microbiota rapidly from the mother or surrounding environment after birth (Evans et al., 2016; Go et al., 2016; Caprara et al., 2024). However, some research indicates that perhaps during the embryonic period, infants may have already been exposed to microbial communities (Aagaard et al., 2014). Regardless, throughout a person’s life, the gut microbiota dynamically changes and impact our physiological conditions.

The gut microbiota produces a wide array of metabolites, including bile acids, short-chain fatty acids (e.g., propionate and butyrate), and amino acids, which modulate insulin sensitivity and maintain systemic metabolic homeostasis (Liu et al., 2022c).

### 3.1 Bacteroidetes as a co-biomarker in T2DM, AD and PCOS

A shared feature across T2DM, AD, and PCOS is insulin resistance, which is increasingly linked to gut microbiota dysbiosis. In T2DM, a classic metabolic disorder characterized by such dysbiosis, a reduction in the abundance of *Bacteroides* and Bifidobacterium, alongside an increase in *Fusobacterium*, is commonly observed (Ma et al., 2019; Gurung et al., 2020). Crucially, this microbiota imbalance is not merely an association but may be causative, as gut microbiota-induced insulin resistance has been identified as a promoter of PCOS development, with *Bacteroides* specifically highlighted as a key microbial biomarker in this context (Liu et al., 2023; Yang YL. et al., 2021). Extending beyond peripheral metabolism, the gut-brain axis provides a pathway through which gut dysbiosis influences central nervous system disorders. Growing evidence links AD to alterations in gut microbiota. While diabetes can exacerbate AD progression via mechanisms like impaired glucose metabolism, gut dysbiosis itself plays a pivotal role, with shifts in *Bacteroides* abundance being a focal point of investigation in AD pathogenesis (Zhang et al., 2023). Thus, the consistent alteration of *Bacteroides* across these three conditions underscores its potential as a common gut microbial biomarker within the shared pathophysiology of metabolic-neuroendocrine dysfunction.

#### 3.1.1 Bacteroidetes related to T2DM

In recent years, Japanese researchers have demonstrated in zebrafish that intestinal dysbiosis is linked to the pathogenesis of T2DM (Okazaki et al., 2019), a finding subsequently corroborated in rat models (Peng et al., 2019). Collectively, these studies indicate that the gut microbiota and its metabolites play a pivotal role in the initiation and progression of T2DM.

Concurrently, the composition and abundance of gut microbes in T2DM patients are markedly altered. Metagenomic analyses by Junjie Qin et al. revealed that, although changes within the phylum Firmicutes are heterogeneous, butyrate-producing genera—such as Faecalibacterium, Roseburia, and Coprococcus—are significantly reduced, whereas Proteobacteria expanded and Bacteroidetes decreased (Qin et al., 2012), The number of Bifidobacterium (Li et al., 2023) decreased, while randomized studies suggested an increase in *Bacteroides* (Zhang et al., 2025). These characteristic microbial shifts can serve as biomarkers for monitoring T2DM development.

#### 3.1.2 Bacteroidetes related toAD

Recent scholarly investigations indicated that dysbiosis of the gut microbiota may facilitate the aggregation of amyloid-β, neuroinflammatory responses, oxidative stress, and insulin resistance, which are implicated in the pathogenesis of AD (Vogt et al., 2017). Different bacterial genera and species can generate various metabolites, such as aminobutyric acid (GABA), serotonin (5-HT), histamine, and dopamine, etc., which are implicated in a series of emotions, behaviors, and cognitive functions as neurotransmitters or precursors of neurotransmitters (Di Benedetto et al., 2017). In the metabolic process, the host and its gut microbiota jointly produce a series of metabolites, such as short-chain fatty acids, which can directly or indirectly mediate microbiota-gut-brain interactions, which is vital to the health of the host (Chen et al., 2021). Intestinal flora dysbiosis induces a reduction in beneficial substances such as short-chain fatty acids (SCFAs) and hydrogen (H_2_), alongside an increase in harmful substances like amyloid and trimethylamine N-oxide (TMAO). This imbalance leads to increased permeability of both the intestinal mucosal barrier and the blood-brain barrier, activates the peripheral immune response, and elevates peripheral and central oxidative stress (OS) levels. Finally, intestinal disorders promote the pathological progression of AD by increasing amyloid plaque formation, neuroinflammation and IR (Vogt et al., 2018). Mouse models have confirmed that the altered gut microbiota in AD is characterized by an expansion of Proteobacteria and Bacteroidetes, along with a marked decrease in Firmicutes—especially Bifidobacterium (Manfredi et al., 2025).

#### 3.1.3 Bacteroidetes related to PCOS

As a prevalent metabolic disorder, PCOS is intrinsically characterized by insulin resistance, hepatic steatosis, and chronic low-grade inflammation (Dumesic et al., 2015). Consequently, its pathogenesis mirrors that of another metabolic disease:T2DM, and is likewise shaped by the gut microbiota. In women with PCOS, the gut microbial profile exhibits a marked increase in the phylum Bacteroidetes; within this phylum, *Bacteroides* species attenuate short-chain fatty acid (SCFA) production, thereby fostering obesity and insulin resistance (Kelley et al., 2016). Notably, model-based comparative analyses have further documented a significant decrease in the abundance of *Lactobacillus* and Bifidobacterium (Zhang et al., 2019).

**FIGURE 2:**
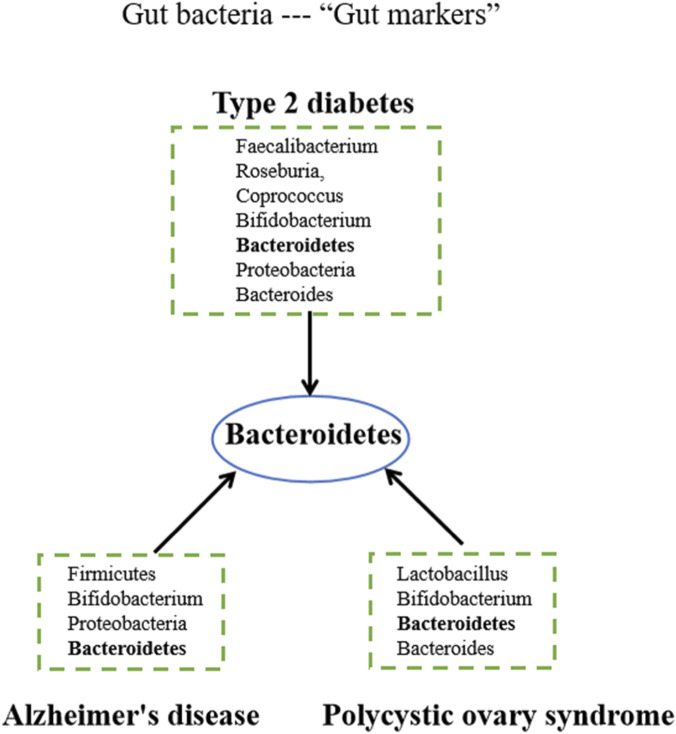
The co-“gut marker” among the three comorbidities.

Based on these findings, we constructed a gut microbial marker map for the comorbidity of T2DM, AD, and PCOS (seen Figure 2), highlighting the consistent reduction of *Bacteroides* across all three conditions. While the primary drivers of this dysbiosis may differ among diseases:ranging from direct metabolic derangement in T2DM to brain-gut axis signaling in AD, emerging intervention studies suggest that the resulting microbial shift itself may actively contribute to a shared physiopathologic mechanism. Crucially, a fecal microbiota transplantation study demonstrated that transferring gut microbiota from AD patients to animal models exacerbated cognitive decline and neuropathology, directly proving the pathogenic potential of disease-associated dysbiosis (Jia et al., 2025). Furthermore, a large-scale meta-analysis of gut microbiomes across multiple diseases revealed a significant compositional similarity between AD and type 2 diabetes, underscoring the existence of a common microbial basis for these comorbidities (Jin DM. et al., 2024). Therefore, the consistent alteration of *Bacteroides* highlighted in our Figure 2 may represent more than a correlative biomarker; it may reflect a common, functionally consequential microbial disturbance that actively participates in the intertwined pathophysiology of metabolic and neuroendocrine disorders. This perspective strengthens the rationale for investigating *Bacteroides* not only as a diagnostic marker but also as a potential therapeutic target for modulating the shared disease susceptibility underlying T2DM, AD, and PCOS.

## 4 Therapeutic effects of DSS and its principal blood-entry constituents

Clinically, DSS has demonstrated significant therapeutic outcomes in managing T2DM, AD, and PCOS, supporting its role as a shared therapeutic agent. For T2DM, clinical studies confirm that DSS not only improves glycemic control but also alleviates specific complications, including diabetic neuropathy and nephropathy, suggesting a systemic protective effect beyond mere glucose-lowering (Xiao-bin, 2019; Material et al., 2022). In AD, DSS intervention is associated with improved cognitive function in patients, as evidenced by clinical evaluations and meta-analyses; this benefit is likely mediated through modulation of brain insulin signaling, neuroinflammation, and Aβ metabolism (Haifang, 2023; Kim and Cho, 2020). Furthermore, in PCOS management, DSS has been shown to ameliorate core pathological features, including insulin resistance and reproductive endocrine dysfunction, leading to reduced hyperandrogenism, improved ovarian function, and restored menstrual cyclicity (Zhang and shelley, 2023; Yang et al., 2020). Collectively, these convergent clinical and preclinical findings across three distinct disease domains underscore the capacity of DSS to target shared pathophysiological axes, particularly insulin resistance and neuroendocrine imbalance thereby validating its multi-target, multi-disease therapeutic potential.

Therefore, a detailed examination of the mechanisms by which the major blood-entry components of DSS exert their effects—through regulation of insulin signaling, inflammatory responses, and other shared pathways—is presented in the following sections.

As key botanical drugs of DSS, both Angelica sinensis and Ligusticum chuanxiong are rich in phthalides. The characteristic dimeric phthalide, Riligustilide (RG), along with its monomeric precursor Z-ligustilide, are considered major bioactive contributors. Experimental evidence confirms RG’s direct actions in T2DM management, such as activating PPARγ and insulin signaling to enhance insulin sensitivity and attenuate gluconeogenesis (Qu et al., 2022). However, the pharmacokinetic profile of such phthalides presents a complexity: the parent form of RG may exhibit limited oral bioavailability due to chemical instability and first-pass metabolism, making its direct detection in circulation challenging (Zhang Y. et al., 2020). Importantly, these constituents can be absorbed and extensively metabolized (Xie et al., 2020). Thus, the therapeutic contribution of Angelica and Chuanxiong in DSS likely involves not only providing RG but also, through herb-herb interactions, promoting the bioavailability of active phthalides or utilizing their metabolites to jointly modulate pathological pathways.

The active constituent derived from *Angelica sinensis*, a principal botanical drug in the formula, can ameliorate memory impairment by modulating neurotransmitter balance, free-radical metabolism, inflammation, and neuronal apoptosis, and by activating the BDNF/TrkB/CREB pathway to regulate the onset and progression of AD (Du et al., 2020). Meanwhile, ferulic acid, a key bioactive phenolic acid present in *A. sinensis* (and other DSS herbs), modulates multiple metabolic pathways—such as PI3K/AKT, PPAR, MAPK, AMPK, and the insulin signaling pathway—to regulate glucose–lipid metabolism and hormone levels, thereby contributing to the alleviation of PCOS (Gao et al., 2023).

**FIGURE 3:**
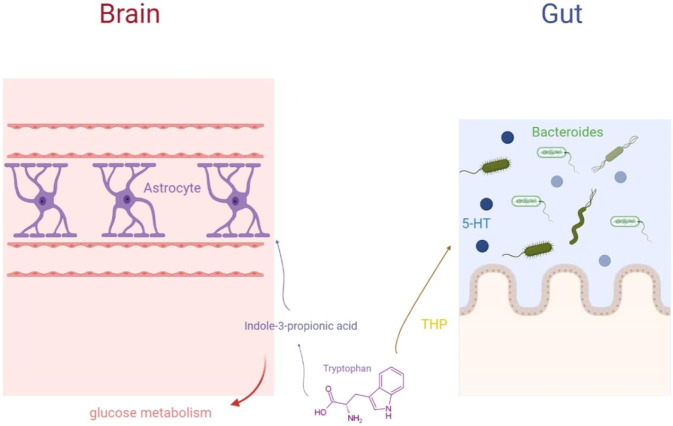
Tryptophan-Bacteroides-Brain-Gut.

Emerging evidence indicates that the gut-brain axis orchestrates bidirectional communication through microbial-host co-metabolism, wherein commensal *Bacteroides* convert dietary tryptophan into neuroactive metabolites such as indole-3-propionic acid (IPA) and serotonin (5-HT). IPA exhibits neuroprotective properties, while 5-HT modulates synaptic transmission and cognitive processes (Gao et al., 2020). Critically, this positions specific *Bacteroides* species or their metabolic pathways as potential therapeutic targets. Modulating their abundance or activity to enhance the production of IPA and 5-HT could represent a novel strategy for improving neuronal function. Concurrently, astrocytic regulation of glucose metabolism provides essential energy substrates for neuronal activity, and tetrahydroprogesterone (THP)-mediated pathways further integrate tryptophan catabolism with nitrogen homeostasis and metabolic water production (Magistretti et al., 2018). As shown in Figure 3, this interconnected network underscores the tripartite interplay between gut microbiota (particularly *Bacteroides*), central neurochemistry, and brain energy homeostasis, highlighting actionable targets within the gut microbiome for neurological disorders.

### 4.1 Principal blood-entry constituents of DSS

As a traditional Chinese formula, DSS exerts its effects through multiple bioactive constituents, multiple targets, and multiple therapeutic effects. While compounds that reach the systemic circulation are crucial for efficacy (Yu et al., 2025), it is essential to distinguish their origin. Chemical fingerprinting and pharmacokinetic analyses have identified several key blood-entry components, which are primarily plant-derived bioactive compounds absorbed from the herbal formulation. Among them, five have been consistently detected and are of major interest: Angelica polysaccharide (from Angelica sinensis), paeoniflorin (from Paeonia lactiflora), tetramethylpyrazine (ligustrazine, from Ligusticum chuanxiong), senkyunolide I (from Ligusticum chuanxiong), and ferulic acid (from Angelica sinensis and Ligusticum chuanxiong) (Huang et al., 2025; Guoqiang et al., 2017). Elucidating the pharmacological roles of these absorbed components helps explain the efficacy of DSS against AD, T2DM, and PCOS. Based on these findings, the following sections detail their mechanisms of action.

#### 4.1.1 Blood-entry constituents and tryptophan

Angelica sinensis polysaccharide, the main active constituent of Angelica, exhibits anti-inflammatory (Du et al., 2020), antioxidant, anti-apoptotic (Du et al., 2023), and hepatoprotective (Wen et al., 2022) effects, which are likely mediated by the modulation of specific signaling pathways. Zhou et al. demonstrated that Angelica polysaccharide exerts anti-inflammatory effects by inhibiting the TLR4/MyD88/NF-κB signaling pathway, and notably, the serum metabolite 5-methyl-dl-tryptophan (5-MT) was restored by Angelica polysaccharide treatment (Zou et al., 2023). Paeoniflorin, another major blood-entry metabolite of DSS, has also been shown to activate the TRPA1 channel and the PLC-γ1/PIP2 signaling pathway to promote 5-HT release, thereby modulating tryptophan metabolism (Zhan et al., 2021). Additionally, paeoniflorin was reported to reduce the expression of the L-tryptophan-catabolizing enzyme tryptophan-2,3-dioxygenase in the liver of stress-induced depressive mice, thereby increasing the 5-hydroxytryptamine/tryptophan ratio and decreasing the kynurenine/tryptophan ratio (Liang et al., 2023). Tetramethylpyrazine (ligustrazine), an alkaloid and one of the main metabolites of Ligusticum chuanxiong in DSS, possesses diverse physiological functions, including antioxidant, anti-inflammatory, anti-apoptotic, autophagy modulation, vasodilation, angiogenesis regulation, mitochondrial damage inhibition, endothelial protection, and neuroprotection (Lin et al., 2022). Studies have found that ligustrazine can modulate the mRNA expression levels of tryptophan and 5-hydroxytryptamine indoleacetic acid, increase 5-HT concentration, and exert anxiolytic-like effects (Lee et al., 2018). Senkyunolide I, a natural phthalide widely distributed in umbelliferous plants, exhibits analgesic, anti-inflammatory, antioxidant, and antithrombotic pharmacological effects (Huang et al., 2023). Notably, senkyunolide I is also considered to alter levels of 5-hydroxytryptamine (5-HT), 5-hydroxytryptophan (5-HTP), 5-hydroxyindoleacetic acid (5-HIAA), norepinephrine (NE), and dopamine (DA) in blood and brain, thereby exerting analgesic and anti-migraine effects (Wang et al., 2011).

#### 4.1.2 Blood-entry constituents and phenylalanine

Ferulic acid, an active constituent widely found in traditional Chinese medicines such as Angelica sinensis and Ligusticum chuanxiong, exhibits biological activities in oxidative stress, inflammation, vascular endothelial injury, fibrosis, apoptosis, and platelet aggregation (Li et al., 2021). Since ferulic acid is derived from the metabolism of phenylalanine and tyrosine (Jin P. et al., 2024), changes in blood ferulic acid levels are accompanied by corresponding alterations in phenylalanine. Apart from ferulic acid, paeoniflorin is also involved in phenylalanine metabolism, such as modulating phenylalanine metabolism in rats with rheumatoid arthritis (Liu et al., 2022d) and endometriosis (Wu et al., 2019), and restoring biomarker levels (phenylalanine). Yuan et al. employed metabolomics combined with network pharmacology to reveal that ligustrazine can regulate phenylalanine metabolism in rats with neuropathic pain, indicating a close relationship between ligustrazine and phenylalanine (Yuan et al., 2025).

#### 4.1.3 Blood-entry constituents and bacteroidetes

Currently, studies on the relationship between blood-entry constituents and Bacteroidetes are scarce. Tang et al. reported that the abundance of Bacteroidetes in type 2 diabetic mice could be restored by Angelica polysaccharide, and the Firmicutes/Bacteroidetes ratio could also be reversed following Angelica polysaccharide treatment (Tang et al., 2023). Similar findings were observed with paeoniflorin, where administration of paeoniflorin to rats with irritable bowel syndrome significantly restored the Firmicutes/Bacteroidetes ratio (Wang et al., 2023).

## 5 Discussion

DSS, a classical multi-herbal formula, demonstrates therapeutic potential across three distinct disorders: T2DM, Alzheimer’s disease (AD), and PCOS. This review posits that these broad effects can be conceptualized as a Shared Therapeutic Approach, underpinned by DSS’s ability to modulate a convergent pathological network common to all three conditions. Our integrative analysis identifies the dysregulation of plasma tryptophan and phenylalanine, coupled with a consistent alteration in the gut bacterial genus *Bacteroides*, as core components of this network. This triad forms a functionally interconnected axis within the gut-brain ecosystem, linking peripheral metabolic and inflammatory states to central nervous system and endocrine functions. The co-occurrence of these biomarkers in T2DM, AD, and PCOS suggests they represent a shared pathophysiological substrate, which may explain the overlapping clinical features such as insulin resistance.

The proposed Shared Therapeutic Approach is mechanistically grounded in DSS’s capacity to act as a multi-target system. Its efficacy likely stems from the synergistic actions of its diverse bioactive constituents rather than a single compound. For instance, absorbed small molecules may directly correct host metabolic and inflammatory pathways. Simultaneously, other components, such as polysaccharides, may function as prebiotics to restore a beneficial gut microbial ecology, including *Bacteroides* populations. This rebalanced microbiota can subsequently aid in normalizing tryptophan and phenylalanine metabolism. Thus, DSS exemplifies a dual-track strategy that simultaneously targets both host pathways and the gut microbiome to rectify the shared network, thereby addressing diverse clinical endpoints from a common root.

It is crucial to acknowledge the limitations within the current evidence supporting this approach. First, while correlative data are compelling, definitive causal evidence establishing that DSS’s clinical benefits are directly mediated through the normalization of this specific biomarker network requires further validation. Second, the pharmacological complexity of DSS,where in the synergistic interactions among its numerous constituents are still not fully mapped, poses a significant challenge for mechanistic reductionism and standardization. Finally, there is a notable gap in direct clinical evidence evaluating DSS in patient populations with the specific comorbidities of T2DM, AD, and/or PCOS, which is the ultimate context for validating a shared therapeutic strategy.

To translate this conceptual framework into an evidence-based paradigm, future research should prioritize several directions. Longitudinal clinical studies in comorbid populations are essential to correlate modulation of the tryptophan-phenylalanine-Bacteroides axis with clinical outcomes. Causal experimental models are needed to validate the indispensable role of this network. Furthermore, applying integrated multi-omics analyses to DSS intervention studies will help delineate the comprehensive biological network it influences.

## 6 Conclusion

This review summarizes and synthesizes emerging evidence supporting the role of DSS in T2DM, AD, and PCOS through shared biomarker networks. The consistent involvement of tryptophan, phenylalanine, and *Bacteroides* across these comorbidities provides a novel, TCM-inspired perspective on their interconnected pathogenesis. While these associations highlight the potential of DSS as a multi-target agent, future studies should prioritize clinical validation of these mechanisms in human cohorts and further explore the synergistic actions of DSS metabolites. Such work will be crucial for translating the concept of “a shared therapeutic approach” into evidence-based practice.
