# Supplementary material for: Metabolite and gut microbiota co-biomarkers in Danggui Shaoyao San: insights into a shared therapeutic approach
Source: Front Pharmacol. 2026 Jan 12;16:1698734. doi: 10.3389/fphar.2025.1698734 (PMC12833331; doi:10.3389/fphar.2025.1698734)
Supplement: Supplementary file 1 [file Table1.pdf]

Supplement table1

	Angelica sinensis	*Paeonia lactiflora*	*Alisma plantago-aquatica*	*Atractylodes macrocephala*	*Ligusticum sinense 'Chuanxiong'*
Roots and rhizomes	The main root is thick and fleshy, cylindrical or conical in shape, with a surface ranging from yellow brown to dark brown, and longitudinal wrinkles and transverse pores. The roots have a strong aroma	The fleshy tuberous root is thick and robust, spindle shaped or cylindrical, with a light brown to yellow white skin and a white or light pink interior, with obvious longitudinal wrinkles.	The underground tuber is spherical or irregular nodular, 1-4.5 cm in diameter, with brown skin and dense fibrous roots, and the interior is white or light yellow	The root and stem are enlarged, irregularly shaped or fist-shaped, with a fragrant smell and a sweet and slightly pungent taste.	The rhizome is irregular nodular fistula, the surface is yellowish brown to dark brown, densely covered with annular rings, and has a strong and special aroma.
stem	Upright, hollow, with longitudinal edges on the surface, usually purple in color, with multiple branches on the upper part of the stem, and covered with short hairs when young.	Straight, cylindrical, with a woody base, smooth or with fine ridges on the surface, and many branches at the top.	Straight, hollow, smooth or with fine ridges on the surface, and woody at the base	Straight, cylindrical, green or purplish on the surface, branched at the top, woody at the base. The middle and lower parts of the stem are often covered with spider-like soft hairs.	Straight, cylindrical, hollow, with longitudinal ridges on the surface and obvious nodes. The upper part of the stem is branched and slightly purple when young.
leaf	The basal and stem leaves are both compound leaves with two to three lobes and three lobes, and the leaves are ovate to broadly ovate with serrated edges. The base of the petiole swells into a sheath like shape, enveloping the stem	Bipinnate compound leaf, 9-12 leaflets, ovate to lanceolate, 5-12 cm long, 2-5 cm wide, entire margin, dark green on the surface, gray-green on the back. Leaf sequence: alternate, with sheath-like petiole at the base.	The basal leaves are clustered and have long petioles (up to 50 cm), which are enlarged into a sheath at the base; the leaf morphology is diverse: submerged leaves: linear or lanceolate; emergent leaves: broad lanceolate, elliptical to ovate, 2-18 cm long, 1.3-10 cm wide, with 5-7 veins and entire margins	Mutual growth, leathery leaves, ovate-lanceolate to elliptical, 3-15 cm long, 1.5-6 cm wide, margins with spiny teeth or pinnately deeply divided (basal leaves mostly lobed, upper leaves entire). Veins: the main vein prominent, lateral veins reticulate, leaf surface dark green, underside grayish-green, densely covered with white down. Petiole: 0.5-4 cm long, base semi-clasping the stem.	Basal and stem leaves are bipinnate to tripinnate compound leaves, with 3-5 pairs of leaflets. The leaf is ovate-lanceolate, with deeply pinnate or entire margins. The petiole expands into a sheath at the base, embracing the stem,

flower	Umbelliferae inflorescence terminal or lateral, inflorescence diameter 5-15 cm, small flowers white or light green.	Single flowers are borne at the top of the stem or branch, and the flowers are large and gorgeous, with a diameter of 8-15 cm.	Refractory panicle inflorescence, with 3-8 branches, each wheel 3-9; floral structure: outer whorl of sepals 3, ovate, green or purple; inner whorl of sepals 3, nearly round, white, pink or light purple	The inflorescence is terminal, 2-4 cm in diameter, with 5-7 layers of bracts. The outer bracts are ovate and the inner bracts are lanceolate with membranous margins.	The compound umbellate inflorescence is terminal or lateral, the small flowers are white, with 5 petals and the apex is curled inward.
fruit	Double hanging fruit flat, elliptical, winged on both sides, splits into two separate fruits when mature.	The follicle is ovoid, 2-3 cm long, densely covered with villi on the surface, and splits along the ventral suture when mature.	The achenes are flat, ellipsoidal or nearly rectangular, about 2.5 mm long, with 1-2 shallow grooves on the back, and brown when mature	Achenes: elongated ellipsoidal, about 8 mm long, densely covered with white long soft hairs, the ciliate is feathered, 1-1.5 cm long, grayish white.	The double fruit is ovoid, slightly flattened on both sides, with 5 ribs in the division, and the fruit period is from August to September.

Note: The information comes from Zhiwu Zhi (www.iplant.cn).
