# Supplementary material for: Metabolite and gut microbiota co-biomarkers in Danggui Shaoyao San: insights into a shared therapeutic approach
Source: Front Pharmacol. 2026 Jan 12;16:1698734. doi: 10.3389/fphar.2025.1698734 (PMC12833331; doi:10.3389/fphar.2025.1698734)
Supplement: Supplementary file 2 [file Table2.pdf]

Supplement table 2

the common biological markers for T2DM

Metabolite/Biomarker	Location	Change in T2DM Patients	Reference
Adipsin	Serum	↓	[1]
Visfatin	Serum	↑	[1]
Nesfatin-1	Plasma	↑	[2]
Netrin-1	Serum	↑	[3]
VCAM-1	Serum	↑	[3]
Protein Z (PROZ)	Plasma	↓	[4]
Neuregulin-4	Serum	↑	[5]
Mitochondrial DNA (mtDNA) fragments (ND4, Cox1, D-loop)	Plasma	↑	[6]
HMGB1	Plasma	↑	[7]
Wnt5a	Serum	↓	[8]
Haptoglobin glycated K141 (HP K141)	Plasma (glycation site)	↑	[9]
HbA_{1c}	Erythrocytes (Hemoglobin)	↑	[7-9]
Glycine	Plasma	↓	[10, 11]
Taurine	Plasma	↓	[10]
Phenylalanine	Plasma	↑	[10-13]
Valine	Plasma，Serum	↑	[11-14]
Isoleucine	Plasma，Serum	↑	[11-14]
Glutamic acid	Serum	↑	[11]
Proline	Plasma，Serum	↑	[11-13]
Alanine	Serum	↑	[11, 12]12， 14
Lysine	Plasma，Serum	↑	[11, 13]
Leucine	Plasma，Serum	↑	[11-14]
Tyrosine	Serum	↑	[11, 12]
Ornithine	Serum	↑	[11]
Tryptophan	Plasma，Serum	↑	[11, 15]
Histidine	Plasma，Serum	↑	[11, 13]
Methionine	Serum	↑	[11]
Serine	Serum	↓	[11]
Glutamine	Serum	↓	[11, 12]
Asparagine	Serum	↓	[11]
Pyroglutamic acid	Plasma	↓	[12]
Aminobutyric acid	Plasma	↑	[12]

Hydroxyisobutyric acid	Plasma	↑	**[12]**
Acetylcarnitine (C2)	Plasma	↑	**[12]**
Propionylcarnitine (C3)	Plasma	↓	**[12]**
Dodecenoylcarnitine	Plasma	↑	**[12]**
Tetradecenoylcarnitine	Plasma	↑	**[12]**
Hexadecadienoylcarnitine	Plasma	↓	**[12]**
Oleylcarnitine	Plasma	↓	**[12]**
Butyryl/2-Methylbutyryl (C4)	Plasma (Pediatric IR)	↑	**[15]**
Palmitic acid (16:0)	Plasma	↑	**[12]**
Stearic acid (18:0)	Plasma	↓	**[12]**
Linoleic acid (18:2n6)	Plasma	↑	**[12]**
Arachidonic acid (20:4n6)	Plasma	↑	**[12]**
Oleic acid (18:1n9)	Plasma	↑	**[12]**
Docosahexaenoate (DHA)	Plasma (Pediatric IR)	↓	**[15]**
Sphingomyelin (d18:2/16:0)	Plasma	↓	**[12]**
Taurodeoxycholate	Plasma (Pediatric IR)	↑	**[15]**
Glycodeoxycholate	Plasma (Pediatric IR)	↑	**[15]**
3-Hydroxybutyrate	Plasma	↓	**[12]**
Pyruvate	Plasma	↑	**[12]**
Lysophospholipids (e.g., LPC, LPE)	Plasma (Pediatric IR)	↑	**[15]**
Saturated fatty acids	Plasma	↑	**[13]**
3-Hydroxybutyric acid	Plasma	↑	**[13]**
Lactate	Plasma	↑	**[13]**
Choline	Plasma	↑	**[13]**
3,7-Dimethyluric acid	Plasma	↑	**[13]**
Pantothenic acid	Plasma	↑	**[13]**
Myoinositol	Plasma	↑	**[13]**
Threonine	Plasma	↑	**[13]**
Sorbitol	Plasma	↑	**[13]**
Glycerol	Plasma	↑	**[13]**
Glucose	Plasma	↑	**[13]**
LPC C16:0	Plasma	↑	**[16]**
PC C16:0/18:0	Plasma	↓	**[16]**

Supplement table 3

the common biological markers for AD

Metabolite/Biomarker	Location	Change in AD Patients	Reference
Neurofilament Light (NfL)	Plasma	↑	**[17-22]**

Phosphorylated Tau 181 (p-tau181)	Plasma	↑	**[19-21, 23, 24]**
CSF p-tau181	Cerebrospinal Fluid	↑	**[19]**
CSF Aβ_{1-42}	Cerebrospinal Fluid	↓	**[19, 25]**
Aβ42/40 ratio	Plasma	↓	**[21, 22]**
P-tau217	Plasma	↑	**[22, 23]**
Glial Fibrillary Acidic Protein (GFAP)	Plasma	↑	**[20, 21, 26]**
Total Tau (T-tau)	Plasma	↑	**[21, 24]**
Aβ_{1-42}/Aβ_{1-40} ratio	Plasma	↓	**[25]**
Aβ42	Plasma	↑	**[24, 27]**
α-Synuclein	Plasma	↑	**[24]**
GFAP	Cerebrospinal Fluid (CSF)	↑	**[26]**
Phosphatidylcholines (PCs)	Plasma & Brain	↓	**[28, 29]**
PC aa C36:6, C38:0, C38:6, C40:1, C40:2, C40:6; PC ae C40:6	Plasma	↓	**[28]**
Acylcarnitines (ACs)	Plasma	↓	**[28]**
Propionyl AC (C3), C16:1-OH AC	Plasma	↓	**[28]**
Lysophosphatidylcholine (LysoPC)	Plasma	↓	**[28-30]**
LysoPC(18:0), LysoPC(18:1), LysoPC(20:3-20:5), LysoPC(22:6), LysoPC(16:1)	Plasma	↑	**[29, 31]**
Phosphatidylinositol	Plasma	↓	**[28]**
LysoPE(24:6), LysoPE(22:0), LysoPE(20:5)	Plasma	↓	**[29]**
LysoPE(22:5)	Plasma	↑	**[31]**
Ceramides (Cers)	Plasma	↑	**[29, 31]**
Glycosphingolipids	Plasma	↓	**[29]**
Glycosphingolipids	Brain	↑	**[29]**
Diacylglycerols (DGs)	Plasma & Brain	↑	**[29]**
Triacylglycerols (TGs)	Plasma	↑	**[29]**
Free Fatty Acids	Plasma & Brain	↓	**[29, 31]**
Glycoursodeoxycholic acid	Plasma	↑	**[28]**
Cholic Acid (CA)	Plasma	↓	**[32, 33]**
Taurocholic Acid (TCA)	Brain	↓	**[32, 33]**
Deoxycholic acid (DCA)	Plasma	↑	**[33]**
Lithocholic acid (LCA)	Plasma	↑	**[33]**
Bile Acids (Total)	Plasma	↑	**[34]**
Chenodeoxycholic Acid (CDCA)	Plasma	↑	**[34]**
Allocholic Acid	Plasma	↑	**[34]**
Oxysterols	Plasma	↑	**[29]**
Neuronal Pentraxin 1 (NP1)	Plasma	↑	**[35]**
Brain-Derived Neurotrophic Factor (BDNF)	Plasma	↓	**[36]**

Agmatine	Plasma	↑	**[37]**
Putrescine	Plasma	↓	**[37]**
Spermidine	Plasma	↓	**[37]**
Spermine	Plasma	↓	**[37]**
$A\beta_{1-42}$	Plasma Neuronal-Derived Exosomes (NDEs)	↑	**[38]**
Neurogranin, Synaptophysin, Synaptotagmin, Synaptopodin	Plasma NDEs	↓	**[38]**
BACE activity	Plasma	↑	**[27]**
sAPPβ, sAPPα	Plasma	↑	**[27]**
D-Glucose, D-Galactose	Plasma	↑	**[30]**
Glutathione	Plasma	↓	**[30]**
L-Carnitine, L-Acetylcarnitine	Plasma	↓	**[30]**
Glycerophosphocholine	Plasma	↑	**[31]**
Phosphorylcholine	Plasma	↑	**[31]**
L-Tryptophan	Plasma	↑	**[31]**
Tryptophan	Plasma, CSF	↓	**[39]**
Kynurenine	CSF	↑	**[39]**
Kynurenine/Tryptophan ratio	CSF	↑	**[39]**
Quinolinic acid (QA)	CSF	↑	**[39]**
Picolinic acid (PIC)	CSF	↓	**[39]**
Neopterin (NEO)	CSF	↑	**[39]**
Ceramide (Cer)	CSF	↑	**[39]**
Sphingomyelins (SM)	CSF	↓	**[39]**
Ceramide/Sphingomyelin ratio	CSF	↑	**[39]**
Phosphatidylcholines (PC)	CSF	↓	**[39]**
Polyunsaturated Fatty Acids (PUFAs)	Plasma	↓	**[34]**
Acyl-carnitines (medium/long chain)	Plasma	↓	**[34]**
Indolelactic acid	Plasma	↓	**[34]**
2-Hydroxyisovaleric acid	Urine	↓	**[40]**
Guanidinoacetate	Urine	↑	**[40]**
Urocanate	Urine	↑	**[40]**
Hippuric acid	Urine	↓	**[40]**
Cytosine	Urine	↑	**[40]**
2-Ketoisovalerate	Urine	↓	**[40]**
Malonate	Urine	↑	**[40]**
Glucose	Urine	↑	**[40]**
Phenylalanine	CSF	↑	**[41]**
Arachidonic acid (ARA)	Plasma	↑	**[41]**
ARA	CSF	↓	**[41]**
Docosahexaenoic acid (DHA)	CSF	↓	**[41]**
Eicosapentaenoic acid (EPA)	CSF	↓	**[41]**
Cortisol	Serum,CSF	↑	**[41]**

Dehydroepiandrosterone (DHEA)	Plasma	↓	**[41]**
Leukotriene B4 (LTB4)	CSF	↑	**[41]**
14,15-DHET	Plasma	↓	**[41]**
Sphingosine	Blood	↓	**[41]**
LysoPC species	Plasma	↑	**[41]**

Supplement table 4

the common biological markers for PCOS

Metabolite/Biomarker	Location	Change in PCOS Patients	Reference
Glutamine	Plasma	↓	[42]
Isoleucine	Plasma	↓	[42]
Choline	Plasma	↓	[42]
Sphingomyelin	Plasma	↑	[42]
Free Fatty Acids (Total)	Plasma	↑	[43]
Citrate	Plasma	↓	[42]
Lactate	Plasma	↑	[42]
Creatine	Plasma	↑	[42, 44]
Glutamic acid	Plasma	↑	[45-47]
Phenylalanine	Plasma	↑	[45]
Valine	Plasma	↑	[45]
Tyrosine	Plasma	↑	[45]
Tryptophan	Plasma	↑	[45]
Ornithine	Plasma	↑	[45]
Free fatty acids (FFAs)	Plasma	↑	[45]
Decanoylcarnitine	Plasma	↑	[48]
Palmitic acid	Follicular fluid,Plasma	↑	[48, 49]
Pentadecanoic acid	Follicular fluid	↑	[49]
Citric acid	Follicular fluid	↑	[49]
Isocitric acid	Follicular fluid,Urine	↑	[49]
Pyruvic acid	Follicular fluid	↑	[49]
Lithocholic acid	Follicular fluid	↓	[49]
Sinapinic acid	Follicular fluid	↓	[49]
5-Methoxysalicylic acid	Serum	↑	[44]
Argininosuccinic acid	Serum	↓	[44]
Dodecanedioic acid	Serum	↓	[44]
Indoxyl sulfate	Serum	↓	[44]
Linoleic acid	Serum	↑	[44]
Myristic acid	Serum	↑	[44]
Palmitoleic acid	Serum	↑	[44]
Vaccenic acid	Serum	↑	[44]
Phenylalanyl-histidine	Urine	↑	[50]
Gamma-glutamyl-leucine	Urine	↓	[50]

Indoleacetyl glutamine	Urine	↓	[50]
Cortolone-3-glucuronide	Urine	↑	[50]
Methylmalonic acid	Urine	↑	[51]
Phosphoric acid	Urine	↓	[51]
Xylitol	Urine	↓	[51]
Xylose	Urine	↓	[51]
Ribitol	Urine	↓	[51]
Glycolic acid	Urine	↓	[51]
4-Hydroxycyclohexanecarboxylic acid	Urine	↑	[51]
Tartronic acid	Urine	↑	[51]
Acylcarnitine C2	Plasma	↓	[52]
1-Methylnicotinamide	Serum	↓	[46]
7β-Hydroxycholesterol	Follicular Fluid	↓	[53]
Acetylcarnitine	Serum	↓	[46]
Aspartic acid	Serum	↓	[46]
CDCA-3β-Gln (Chemodeoxycholic acid-3-β-d-glucuronide)	Follicular Fluid	↑	[54]
GCA (Glycocholic acid)	Follicular Fluid	↑	[54]
GCDCA (Glycochenodeoxycholic acid)	Follicular Fluid	↑	[54]
Glycerophosphocholine	Serum	↑	[46]
L-Carnitine	Follicular Fluid	↑	[53]
Oleamide	Serum	↑	[46]
Pyridoxal 5'-phosphate	Follicular Fluid	↑	[53]
TCA (Taurocholic acid)	Follicular Fluid	↑	[54]
Primary bile acids	Follicular Fluid	↑	[54]
Conjugated bile acids	Follicular Fluid	↑	[54]
Triglycerides (TG)	Follicular Fluid	↑	[55]
Phosphatidylethanolamines (PE)	Follicular Fluid	↑	[55]
Ceramides (Cer)	Serum	↑	[56]
Monomethyl sulfate	Serum	↑	[57]
Riboflavin (Vitamin B_2)	Serum	↑	[57]
Oxoglutaric acid	Serum	↑	[57]
4-Hydroxybenzoic acid	Serum	↑	[57]
N-Acetyldemethylphosphinothricin	Serum	↑	[57]
L-Cysteine	Serum	↑	[57]
LPI(18:0)	Serum	↑	[58]
DHA-containing lipids	Serum	↓	[58]
Branched-chain amino acids (BCAA: Valine, Leucine, Isoleucine)	Plasma	↑	[47]
Aromatic amino acids (Tyrosine, Phenylalanine, Tryptophan)	Plasma	↑	[47]
Threonine	Plasma	↑	[47]
Sarcosine	Plasma	↑	[47]
Citrulline	Plasma	↑	[47]

Asparagine	Plasma	↑	[47]
Histidine	Plasma	↑	[47]
GABA	Plasma	↑	[47]
Alanine	Plasma	↑	[47]
Lysine	Plasma	↑	[47]
3-Methylhistidine	Plasma	↑	[47]
Hydroxyarginine	Plasma	↑	[47]
Glycine	Plasma	↓	[47]
5-Hydroxylysine	Plasma	↓	[47]
Phosphoethanolamine	Plasma	↓	[47]
LBP	Plasma (Serum)	↑	[59]

[1] Legakis I, Mantzouridis T, Bouboulis G, Chrousos GP. Reciprocal changes of serum adispin and visfatin levels in patients with type 2 diabetes after an overnight fast. Arch Endocrinol Metab 2016; 60(1): 76-8.

[2] Xu D, Yu Y, Xu Y, Ge J. Plasma Nesfatin-1: Potential Predictor and Diagnostic Biomarker for Cognitive Dysfunction in T2DM Patient. Diabetes Metab Syndr Obes 2021; 14: 3555-66.

[3] Fadel MM, Abdel Ghaffar FR, Zwain SK, Ibrahim HM, Badr EA. Serum netrin and VCAM-1 as biomarker for Egyptian patients with type II diabetes mellitus. Biochem Biophys Rep 2021; 27: 101045.

[4] Bae YU, You JH, Cho NH, Kim LE, Shim HM, Park JH, et al. Association of Protein Z with Prediabetes and Type 2 Diabetes. Endocrinol Metab (Seoul) 2021; 36(3): 637-46.

[5] Kocak MZ, Aktas G, Erkus E, Yis OM, Duman TT, Atak BM, et al. Neuregulin-4 is associated with plasma glucose and increased risk of type 2 diabetes mellitus. Swiss Med Wkly 2019; 149: w20139.

[6] Yuzefovych LV, Pastukh VM, Ruchko MV, Simmons JD, Richards WO, Rachek LI. Plasma mitochondrial DNA is elevated in obese type 2 diabetes mellitus patients and correlates positively with insulin resistance. PLoS One 2019; 14(10): e0222278.

[7] Wang H, Qu H, Deng H. Plasma HMGB-1 Levels in Subjects with Obesity and Type 2 Diabetes: A Cross-Sectional Study in China. PLoS One 2015; 10(8): e0136564.

[8] Xu W, Geng H, Liu X, Wang X, Li R, Lv Q, et al. Wingless-type MMTV integration site family member 5a: a novel biomarker regulated in type 2 diabetes mellitus and diabetic kidney disease. J Diabetes Metab Disord 2019; 18(2): 525-32.

[9] Spiller S, Li Y, Blüher M, Welch L, Hoffmann R. Glycated lysine-141 in haptoglobin improves the diagnostic accuracy for type 2 diabetes mellitus in combination with glycated hemoglobin HbA(1c) and fasting plasma glucose. Clin Proteomics 2017; 14: 10.

[10] Merino J, Leong A, Liu CT, Porneala B, Walford GA, von Grotthuss M, et al. Metabolomics insights into early type 2 diabetes pathogenesis and detection in individuals with normal fasting glucose. Diabetologia 2018; 61(6): 1315-24.

[11] Okekunle AP, Li Y, Liu L, Du S, Wu X, Chen Y, et al. Abnormal circulating amino acid profiles in multiple metabolic disorders. Diabetes Res Clin Pract 2017; 132: 45-58.

[12] Abu Bakar MH, Sarmidi MR. Association of cultured myotubes and fasting plasma metabolite profiles with mitochondrial dysfunction in type 2 diabetes subjects. Mol Biosyst 2017; 13(9): 1838-53.

[13] Gogna N, Krishna M, Oommen AM, Dorai K. Investigating correlations in the altered metabolic profiles of obese and diabetic subjects in a South Indian Asian population using an NMR-based metabolomic approach. Mol Biosyst 2015; 11(2): 595-606.
[14] Ramzan I, Ardavani A, Vanweert F, Mellett A, Atherton PJ, Idris I. The Association between Circulating Branched Chain Amino Acids and the Temporal Risk of Developing Type 2 Diabetes Mellitus: A Systematic Review & Meta-Analysis. Nutrients 2022; 14(20).
[15] Mastrangelo A, Martos-Moreno G, García A, Barrios V, Rupérez FJ, Chowen JA, et al. Insulin resistance in prepubertal obese children correlates with sex-dependent early onset metabolomic alterations. Int J Obes (Lond) 2016; 40(10): 1494-502.
[16] Zhu C, Liang QL, Hu P, Wang YM, Luo GA. Phospholipidomic identification of potential plasma biomarkers associated with type 2 diabetes mellitus and diabetic nephropathy. Talanta 2011; 85(4): 1711-20.
[17] Illán-Gala I, Lleo A, Karydas A, Staffaroni AM, Zetterberg H, Sivasankaran R, et al. Plasma Tau and Neurofilament Light in Frontotemporal Lobar Degeneration and Alzheimer Disease. Neurology 2021; 96(5): e671-e83.
[18] Benedet AL, Leuzy A, Pascoal TA, Ashton NJ, Mathotaarachchi S, Savard M, et al. Stage-specific links between plasma neurofilament light and imaging biomarkers of Alzheimer's disease. Brain 2020; 143(12): 3793-804.
[19] Clark C, Lewczuk P, Kornhuber J, Richiardi J, Maréchal B, Karikari TK, et al. Plasma neurofilament light and phosphorylated tau 181 as biomarkers of Alzheimer's disease pathology and clinical disease progression. Alzheimers Res Ther 2021; 13(1): 65.
[20] Stocker H, Beyer L, Perna L, Rujescu D, Holleczek B, Beyreuther K, et al. Association of plasma biomarkers, p-tau181, glial fibrillary acidic protein, and neurofilament light, with intermediate and long-term clinical Alzheimer's disease risk: Results from a prospective cohort followed over 17 years. Alzheimers Dement 2023; 19(1): 25-35.
[21] Simrén J, Leuzy A, Karikari TK, Hye A, Benedet AL, Lantero-Rodriguez J, et al. The diagnostic and prognostic capabilities of plasma biomarkers in Alzheimer's disease. Alzheimers Dement 2021; 17(7): 1145-56.
[22] Pereira JB, Janelidze S, Stomrud E, Palmqvist S, van Westen D, Dage JL, et al. Plasma markers predict changes in amyloid, tau, atrophy and cognition in non-demented subjects. Brain 2021; 144(9): 2826-36.
[23] Thijssen EH, La Joie R, Strom A, Fonseca C, Iaccarino L, Wolf A, et al. Plasma phosphorylated tau 217 and phosphorylated tau 181 as biomarkers in Alzheimer's disease and frontotemporal lobar degeneration: a retrospective diagnostic performance study. Lancet Neurol 2021; 20(9): 739-52.
[24] Lin CH, Chiu SI, Chen TF, Jang JR, Chiu MJ. Classifications of Neurodegenerative Disorders Using a Multiplex Blood Biomarkers-Based Machine Learning Model. Int J Mol Sci 2020; 21(18).
[25] Lopez OL, Chang Y, Ives DG, Snitz BE, Fitzpatrick AL, Carlson MC, et al. Blood amyloid levels and risk of dementia in the Ginkgo Evaluation of Memory Study (GEMS): A longitudinal analysis. Alzheimers Dement 2019; 15(8): 1029-38.
[26] Benedet AL, Milà-Alomà M, Vrillon A, Ashton NJ, Pascoal TA, Lussier F, et al. Differences Between Plasma and Cerebrospinal Fluid Glial Fibrillary Acidic Protein Levels Across the Alzheimer Disease Continuum. JAMA Neurol 2021; 78(12): 1471-83.

[27] Wu G, Sankaranarayanan S, Wong J, Tugusheva K, Michener MS, Shi X, et al. Characterization of plasma β-secretase (BACE1) activity and soluble amyloid precursor proteins as potential biomarkers for Alzheimer's disease. J Neurosci Res 2012; 90(12): 2247-58.
[28] Mapstone M, Cheema AK, Fiandaca MS, Zhong X, Mhyre TR, MacArthur LH, et al. Plasma phospholipids identify antecedent memory impairment in older adults. Nat Med 2014; 20(4): 415-8.
[29] Zhang X, Liu W, Zan J, Wu C, Tan W. Untargeted lipidomics reveals progression of early Alzheimer's disease in APP/PS1 transgenic mice. Sci Rep 2020; 10(1): 14509.
[30] He WJ, Cao DM, Chen YB, Shi JJ, Hu T, Zhang ZT, et al. Explore of the beneficial effects of Huang-Lian-Jie-Du Decoction on diabetic encephalopathy in db/db mice by UPLC-Q-Orbitrap HRMS/MS based untargeted metabolomics analysis. J Pharm Biomed Anal 2021; 192: 113652.
[31] Sun LM, Zhu BJ, Cao HT, Zhang XY, Zhang QC, Xin GZ, et al. Explore the effects of Huang-Lian-Jie-Du-Tang on Alzheimer's disease by UPLC-QTOF/MS-based plasma metabolomics study. J Pharm Biomed Anal 2018; 151: 75-83.
[32] Pan X, Elliott CT, McGuinness B, Passmore P, Kehoe PG, Hölscher C, et al. Metabolomic Profiling of Bile Acids in Clinical and Experimental Samples of Alzheimer's Disease. Metabolites 2017; 7(2).
[33] Koike S, Miyaji Y, Sano H, Aikawa N, Kai M, Kasahara S, et al. Simultaneous Determination of Five Bile Acids as Potential Biomarkers for Alzheimer's Disease in Mouse Brain and Plasma. Anal Sci 2021; 37(8): 1165-70.
[34] Shao Y, Ouyang Y, Li T, Liu X, Xu X, Li S, et al. Alteration of Metabolic Profile and Potential Biomarkers in the Plasma of Alzheimer's Disease. Aging Dis 2020; 11(6): 1459-70.
[35] Ma QL, Teng E, Zuo X, Jones M, Teter B, Zhao EY, et al. Neuronal pentraxin 1: A synaptic-derived plasma biomarker in Alzheimer's disease. Neurobiol Dis 2018; 114: 120-8.
[36] Sugasini D, Yalagala PCR, Subbaiah PV. Plasma BDNF is a more reliable biomarker than erythrocyte omega-3 index for the omega-3 fatty acid enrichment of brain. Sci Rep 2020; 10(1): 10809.
[37] Bergin DH, Jing Y, Mockett BG, Zhang H, Abraham WC, Liu P. Altered plasma arginine metabolome precedes behavioural and brain arginine metabolomic profile changes in the APPswe/PS1ΔE9 mouse model of Alzheimer's disease. Transl Psychiatry 2018; 8(1): 108.
[38] Winston CN, Goetzl EJ, Baker LD, Vitiello MV, Rissman RA. Growth Hormone-Releasing Hormone Modulation of Neuronal Exosome Biomarkers in Mild Cognitive Impairment. J Alzheimers Dis 2018; 66(3): 971-81.
[39] Yan J, Kuzhiumparambil U, Bandodkar S, Dale RC, Fu S. Cerebrospinal fluid metabolomics: detection of neuroinflammation in human central nervous system disease. Clin Transl Immunology 2021; 10(8): e1318.
[40] Yilmaz A, Ugur Z, Bisgin H, Akyol S, Bahado-Singh R, Wilson G, et al. Targeted Metabolic Profiling of Urine Highlights a Potential Biomarker Panel for the Diagnosis of Alzheimer's Disease and Mild Cognitive Impairment: A Pilot Study. Metabolites 2020; 10(9).
[41] Tan TH, Li SW, Chang CW, Chen YC, Liu YH, Ma JT, et al. Rat Hair Metabolomics Analysis Reveals Perturbations of Unsaturated Fatty Acid Biosynthesis, Phenylalanine, and Arachidonic Acid Metabolism Pathways Are Associated with Amyloid-β-Induced Cognitive Deficits. Mol Neurobiol 2023; 60(8): 4373-95.
[42] Sun L, Hu W, Liu Q, Hao Q, Sun B, Zhang Q, et al. Metabonomics reveals plasma metabolic

changes and inflammatory marker in polycystic ovary syndrome patients. J Proteome Res 2012; 11(5): 2937-46.
[43] Zhao X, Xu F, Qi B, Hao S, Li Y, Li Y, et al. Serum metabolomics study of polycystic ovary syndrome based on liquid chromatography-mass spectrometry. J Proteome Res 2014; 13(2): 1101-11.
[44] Dong F, Deng D, Chen H, Cheng W, Li Q, Luo R, et al. Serum metabolomics study of polycystic ovary syndrome based on UPLC-QTOF-MS coupled with a pattern recognition approach. Anal Bioanal Chem 2015; 407(16): 4683-95.
[45] Zhao X, Feng X, Zhao X, Jiang Y, Li X, Niu J, et al. How to Screen and Prevent Metabolic Syndrome in Patients of PCOS Early: Implications From Metabolomics. Front Endocrinol (Lausanne) 2021; 12: 659268.
[46] Ding X, Deng Y, Wang Y, Xue W, Zhu S, Ma X, et al. Serum metabolomic profiling reveals potential biomarkers in assessing the management of women with polycystic ovary syndrome: a randomized controlled trial. Chin Med J (Engl) 2022; 135(1): 79-85.
[47] Ye Z, Zhang C, Wang S, Zhang Y, Li R, Zhao Y, et al. Amino acid signatures in relation to polycystic ovary syndrome and increased risk of different metabolic disturbances. Reprod Biomed Online 2022; 44(4): 737-46.
[48] Chen YX, Zhang XJ, Huang J, Zhou SJ, Liu F, Jiang LL, et al. UHPLC/Q-TOFMS-based plasma metabolomics of polycystic ovary syndrome patients with and without insulin resistance. J Pharm Biomed Anal 2016; 121: 141-50.
[49] Liu R, Bai S, Zheng S, Zhu X, Zhang Y, Xu B, et al. Identification of the Metabolomics Signature of Human Follicular Fluid from PCOS Women with Insulin Resistance. Dis Markers 2022; 2022: 6877541.
[50] Wang W, Wang S, Tan S, Wen M, Qian Y, Zeng X, et al. Detection of urine metabolites in polycystic ovary syndrome by UPLC triple-TOF-MS. Clin Chim Acta 2015; 448: 39-47.
[51] Zhou W, Hong Y, Yin A, Liu S, Chen M, Lv X, et al. Non-invasive urinary metabolomics reveals metabolic profiling of polycystic ovary syndrome and its subtypes. J Pharm Biomed Anal 2020; 185: 113262.
[52] Ożegowska K, Plewa S, Mantaj U, Pawelczyk L, Matysiak J. Serum Metabolomics in PCOS Women with Different Body Mass Index. J Clin Med 2021; 10(13).
[53] Chen X, Lu T, Wang X, Sun X, Zhang J, Zhou K, et al. Metabolic alterations associated with polycystic ovary syndrome: A UPLC Q-Exactive based metabolomic study. Clin Chim Acta 2020; 502: 280-6.
[54] Yang X, Wu R, Qi D, Fu L, Song T, Wang Y, et al. Profile of Bile Acid Metabolomics in the Follicular Fluid of PCOS Patients. Metabolites 2021; 11(12).
[55] Ban Y, Ran H, Chen Y, Ma L. Lipidomics analysis of human follicular fluid form normal-weight patients with polycystic ovary syndrome: a pilot study. J Ovarian Res 2021; 14(1): 135.
[56] Li J, Xie LM, Song JL, Yau LF, Mi JN, Zhang CR, et al. Alterations of Sphingolipid Metabolism in Different Types of Polycystic Ovary Syndrome. Sci Rep 2019; 9(1): 3204.
[57] Chen J, Zhou Q, Zhang Y, Tan W, Gao H, Zhou L, et al. Discovery of novel serum metabolic biomarkers in patients with polycystic ovarian syndrome and premature ovarian failure. Bioengineered 2021; 12(1): 8778-92.
[58] Mousa A, Huynh K, Ellery SJ, Strauss BJ, Joham AE, de Courten B, et al. Novel Lipidomic

Signature Associated With Metabolic Risk in Women With and Without Polycystic Ovary Syndrome. J Clin Endocrinol Metab 2022; 107(5): e1987-e99.
[59] Liang Z, Di N, Li L, Yang D. Gut microbiota alterations reveal potential gut-brain axis changes in polycystic ovary syndrome. J Endocrinol Invest 2021; 44(8): 1727-37.
